# DEMENTIA CARE FOR RESIDENTS IN ASSISTED LIVING: PERSPECTIVES OF STAFF RESPONDING TO BEHAVIORAL EXPRESSIONS

**DOI:** 10.1093/geroni/igz038.2581

**Published:** 2019-11-08

**Authors:** Debra J Dobbs, Sheryl Zimmerman, Anna Beeber, Paula Carder, Stephanie Miller, Jennifer Hodgkinson, Julia Thorp

**Affiliations:** 1 University of South Florida, Tampa, Florida, United States; 2 Cecil G. Sheps Center for Health Services Research, Chapel Hill, North Carolina, United States; 3 Portland State University Institute on Aging, Portland, Oregon, United States; 4 Cecil G. Sheps Center for Health Services Research, Chapel Hill, United States

## Abstract

Background: An estimated 42% of persons who live in assisted living (AL) have dementia. The majority of these individuals express behaviors (such as agitation, aggression, and wandering) that indicate a mismatch between their ability to cope and demands in the social and physical environment. In some cases, AL staff are able to successfully address those behaviors and in other cases they are not. This study explores behavioral expressions of persons with dementia residing in AL, strategies used to address those behaviors, and residents’ behavioral results, as reported by 251 AL healthcare supervisors across seven states. We also examine what differentiates situations deemed successful from situations that were not successful. Methods: Qualitative interviews conducted with healthcare supervisors revealed cases of successful and unsuccessful strategies for addressing severe/disruptive behavioral expressions of persons with dementia residing in AL. During initial analysis, a data-driven conceptual model was developed to identify common structural domains within and across responses, which ranged from recognizing antecedents to final discharge from the AL community. Additionally, content analysis was applied to identify themes. Results: A minority (<5%) of reports indicated that staff recognized antecedents to behaviors, or noted including residents’ families in addressing behaviors. The majority of both successful and unsuccessful cases referenced the use of medications to address behaviors, and a notable proportion (10%) referenced professional psychiatric assessment. Discussion: Findings suggest the benefit of helping staff identify antecedents of behavioral expressions, and the important role of psychiatric assessment for AL residents who experience agitation, aggression, and similar behaviors.

